# Massachusetts Dental Society

**Published:** 1889-08

**Authors:** 


### Reports of Society Meetings.

### MASSACHUSETTS DENTAL SOCIETY—(*Continued*).

*Thursday, June 6, 1889.—Morning Session.*

### PAPER, "THOROUGHNESS," BY WM. H. ATKINSON, M.D., D.D.S., NEW YORK CITY.

After the reading of his paper, Dr. Atkinson continued: "In place of being the mere demonstration of mechanics, dentistry stands to-day on the pinnacle of science, and exhibits an intelligence and development consonant with the activities of human beings. To be a good dentist you must know all that other people know. You must be a theologian, to begin with,—to be moral; you have to be lawyer, anatomist, and pathologist, and you must have your mental pigeon-holes full of reasoning powers, added to that fulness of human life that makes you think that you are called here for a purpose to bless mankind."

### DISCUSSION.

*Dr. W. X. Sudduth.*—I was much pleased with the paper in that Dr. Atkinson proposes a plan that has been in my mind for several years past. We, in Philadelphia, have formulated a scheme that promises during the summer to take tangible form.

We propose to secure a place where clinical operations can be given in our city, which will also serve as a home where dentists can be taken and entertained in a scientific and literary way. We are, further, going to reorganize the Odontological Society of Pennsylvania on the same basis as laid down by Dr. Atkinson in his paper. Our idea is this: We expect to form within our Society a "Dental Club," the privileges of which will be secured by the payment of an additional fee. The membership fee of the Society will be the same as heretofore in order not to make the fees burdensome to young men; the older members will assume the expense of the "Dental Club" room, which will be a social meeting place, where laboratory and other scientific work can be carried on. It will also include a library, where we expect to accumulate scientific reading matter. This will certainly prove of great benefit to the dentists of our city, as well as outside. After office hours they can go there and see the latest exchanges, talk over scientific matters, and all will tend to our social as well as professional advancement.

*Dr. J. G. W. Werner.*—I would certainly approve any method or scheme that had for its object the performing of clinics that would be of any value, for as I look upon them now they seem to be practically of no benefit. Who is there who has been able to see anything to advantage up-stairs? The great inconvenience of having no table for the operator; the desire of every one to see. Five men, possibly six, are all that can satisfactorily see a dental operation performed at the chair. It is impossible to enjoy a good clinic under such conditions. They are not practicable and of no particular benefit to any one as long as the operator is trying to accommodate a crowd of fifty people.

In order to establish our profession on a broader basis we must establish hospitals. In Europe this has been done. They do a deal of good to the masses at large, and it makes dentistry popular. In our large city to-day there are only two schools that give partially gratuitous services to the public. This is the only claim we may have as philanthropists. What we do in our own private offices is only for money.

Clinics are to me a sort of "post-graduate course," and that is what we need. I know the dentist soon forgets what little he has learned in the dental schools, and a post-graduate course, or scientific course, will aid us greatly, and on a basis of this kind I should be willing to contribute my share; a dental club would certainly fail, for dentists are not social enough to contribute towards a social club. There are other clubs that would give more for the money. The plan proposed appeals to me very forcibly. Why do we not establish a free dental hospital? Our dental colleges are not in a financial condition to do free work. They are not benevolent institutions in that sense. Only the minor operations are done there, and for a majority of the work a compensation is received; and when there are so many poor people who require dental services, it seems to me very urgent that a free dental hospital, to which every respectable dentist should contribute his amount, should be established at once in large cities like Boston. In smaller places, of course, they are not so much needed; but when I think how many medical hospitals there are in this city, and not one dental infirmary or hospital, it seems this is where we ought to begin. That is where you want your clinics, and here you will get the sympathy and respect of the masses.

*Dr. I. J. Weatherby.*—Dr. Werner has referred to the numerous hospitals in the city, impliedly, that they are free: a great many are free to the patients upon the payment of ten, fifteen, and twenty-five dollars a week for the privilege of being treated. Now, I think the dental schools of Harvard and Boston are as generous in their operations and arrangements as are the medical colleges. Certainly, if we should have a free dental hospital,—and it has a beautiful aspect, and would work an excellent end,—there would be a still louder cry among dentists that their patients were being taken from them and operated upon gratuitously. The fact is, we have dentists in the city of Boston who keep a sort of hospital of this kind all the year round,—making teeth for five dollars and filling for almost nothing rather than leave the patient go. We have a sufficient number of them to make a dental hospital almost unnecessary. Then, again, it requires a great deal of money and time in order to carry out the plan as presented by the excellent paper of Dr. Atkinson, and in its fulfilment I should fear a repetition of the experience of the Boston Dental College; there you may see the wives of aldermen, and I don't know how many other people who are well able to pay for dental services. Our profession demands support from the public in so far as it is intelligent in its work, sensible and practical in its operations, and I don't think that a plan of this kind would succeed at all. There is also missing among the profession of Boston that homogeneity that would lead them to support a club of this kind to any great extent. It has its beautiful aspects; but until the Boston dentists "come to the altar," and are thoroughly converted in the propriety and necessity of giving the public at large a larger share of their time, it will not succeed.

The colleges are doing good work. They charge a fee for gold fillings, a very small one for amalgam, and do their work reasonably well. They have failures, but there are failures outside of the college clinics; in these institutions the work done under the supervision of a competent demonstrator is generally very well performed. Whatever will conduce to the good of all generally concerned I shall heartily second, but I do not wish any "will-o'-the-wisp" institutions started which will, in their failure, bring discredit and disadvantage to those engaged therein.

*Dr. George W. Lovejoy.*—I cannot let this opportunity pass without expressing my appreciation of Dr. Atkinson's paper. First, in regard to thoroughness. I think the Executive Committee of this Society has exemplified this in a high degree. Then, I think, the dental home, as proposed by Dr. Atkinson, an excellent idea. It would certainly work well. We could commence on a small scale and make it a place where any dentist could go and get any instrument he wants, or perform any experiment,—the making of a crown, for instance. It would be a sort of "home comfort" for him, and that is what we want for dentists,—home comforts and sociality. In Montreal no liquor is allowed in our club, and its absence is marked by many advantages. There is no doubt that the profession would be very much advanced by the establishment of a dental home.

### DISCUSSION ON DR. PARRAMORE'S PAPER, "STERILIZED SPONGE FOR PULP CAPPING."

*Dr. S. H. Guilford.*—I am very sorry to differ with the essayist, but from my own experience I am compelled to do so. In capping the pulp two or three things have been proven in the past: one is, that in doing so we must not irritate it in any way, either mechanically or medicinally; the second point is, that we dare not produce pressure upon the pulp because it will not stand it; and the third is, we must not allow any space to exist between the pulp and that which overlies it.

We all remember how many years were taken with the idea of capping pulps with oxychloride of zinc. It was used for twenty years, and with apparently good results. However, the pulps died, —partly from the irritation caused by the zinc chloride, and partly from the pressure of the material itself. Its use for this purpose was then discarded; it is used to-day in connection with other materials, but not by itself. In olden times they used to cap nerves by striking up a disk of silver to cover the pulp, but this left an air space. That was proven after a time to be bad practice; we found that the pulp, having the opportunity, forced its way through this opening and was strangulated,—irritated by the ragged edges of the opening and strangulation following. Then, again, it was thought that gutta-percha, being inert, would be good, and it was used for a time. It took only a few years, however, to demonstrate the fact that pressure was produced, or with that cap we had space, and that space resulted in the death of the pulp.

In regard to medicinal agents, those that would chemically irritate would not stand. From these and other failures we learn that the pulp will not tolerate chemical or mechanical irritation. Now for the remedy: We must use some substance as a covering of the pulp that will not produce pressure, and for this we can take some of the different varnishes or resins and coat the pulp with them in such a way that it will afford a good covering for the pulp. Then we can place in something that will not irritate it, and then upon this we can introduce the harder substance. In the use of these resins we have perfect adaptation and no space, and can cap pulps with them with a great deal of success.

In capping there are various methods,—some using varnishes, copal, etc.; others take Canada balsam. Dr. Francis, of New York, a great many years ago, recommended equal parts of Canada balsam and chloroform, dipping into this a piece of letter-paper cut a little larger than the opening into the pulp. Over this he placed oxychloride of zinc. I have used this very successfully, however, substituting Japanese bibulous paper for the letter-paper. In the use of sterilized sponge you put a rough substance upon the nerve, the contact of which will certainly irritate that delicate organ. Then, again, sponge is composed so largely of mineral elements that it is necessarily a gritty substance, and I therefore do not think it is best for a capping.

*Dr. T. H. Parramore*, Hampton, Virginia.—I think that sponge is better than anything else, and my experience has proven it. I first thoroughly sterilize a sponge and place it upon the nerve when I insert the filling. There may be some irritation for a short time, because I have known the tooth to ache for a time after filling; but in practice I could regulate the pain fairly well, and the pulp did not die. At first I thought the pain was caused by the death of the pulp, but I found in refilling the patient complained of sensitiveness to the cutting of excavator as well as to thermal changes. Some of these fillings have been inserted as long as two or three years, and it has been so successful in my hands that I am determined to give it a fair trial, and I think the principle is right. The sponge, as I understand it, protects the nerve against pressure, and again, there is no space between as when we used the caps, about which Dr. Guilford spoke. The sponge is pressed against the pulp and not into it. The pulp, as I understand it, must be irritated before it forms secondary dentine. This method of operating was suggested to me by the use of sterilized sponge in the treatment of ulcers.

A month or two ago I had occasion to fill a lateral incisor for a lad sixteen years of age. I referred to my books, and found a note there that the nerve had been exposed and had seemed irritated. I removed the oxyphosphate, intending to leave some as a protection against thermal changes; but unfortunately the whole of it came away, and I discovered the pulp covered with dentine; I could distinctly see the point where it was exposed before. The sponge had disappeared entirely, and no remains of it could be found. The tooth was not dead, because very sensitive in excavating. There is no trouble further than a little sensitiveness to thermal changes.

Another case was that of a young man, in one of whose teeth the filling had only been inserted about six months. It was a lower lateral. I was going to put a crown on the canine root, and my reason for removing the temporary filling was in order to get more room to operate when the crown was attached. In this case, as in the other, the whole of the oxyphosphate accidentally came away, and showed the nerve in just about the same condition as when I covered it six months before. I refilled it, inserting another piece of sponge, and have not seen him since. His sister, however, was in the office week before last, and stated he had experienced no trouble with it.

As I stated before, it is new practice with me. I do not know whether others are using it or not, but until I have evidence that it is unwise I shall continue to practise it. If it is not as I think it to be I shall certainly discontinue it, and will advise you all.

*Dr. W. S. Elliot*, Hartford, Connecticut.—I was very much pleased with the paper, but the doctor leaves the subject in an unfinished state, inasmuch as he does not give us the philosophy of the action of the sterilized sponge. He speaks very distinctly of the issue of the protoplasmic mass into the pulp cavity, but gives us no instance where there has been a union of the sponge with the protoplasm at all, and if I understand the process of repair, it is through the action and the function of the protoplasmic cells. No matter if there is an issue of protoplasmic mass beyond the point of exposure into the cavity of the tooth, I do not see how secondary dentine can be deposited or organized. This must be beneath the point of exposure. As I understand the use of sterilized sponge, the protoplasm with the sponge acts both mechanically and chemically, and is used up in the new tissues. The doctor does not give us any *rationale*, merely presenting demonstrable facts, which I do not understand. I do not see why any other substance should not do as well. I think the physiology of repair exists beneath the point of exposure, and is governed entirely by the protoplasmic cells which in their function produce the secondary dentine, or rather, not the dentine proper, but merely classification of lime salts. Will the doctor not be a little more explicit and give us a little more knowledge of the action of the sterilized sponge upon the pulp?

*Dr. Parramore.*—In a paper read before the Southern Dental Association I enlarged upon the subject, but in this I have avoided it. I did not care to repeat that theory again, and I did not expect to write an article for this meeting. I promised to give a clinic, but stated I had little time to prepare a paper. My belief was, however, that the sponge invited a deposition of germinal matter into its meshes, there to produce secondary dentine. My theory is, it allows germinal matter to collect at the point of exposure rather than in the body of the pulp.

*Dr. W. S. Elliot.*—It is difficult to accept.

*Dr. Weatherby.*—The paper is a very good one; but while I believe the doctor to be a very truthful man, the idea that sponge will take up protoplasmic matter and become secondary dentine I cannot fully accept. It must be in absolute connection with the pulp, and the process of repair goes on from this organ, and not from the protoplasm in the sponge. I believe he did not state that when he had removed the oxyphosphate fillings he found new dentine had been formed.

*Dr. Parramore.*—Yes, I mentioned one case.

*Dr. Weatherby.*—I have my doubts regarding the physiological results of the operation. I have only to suggest to you a most excellent capping for pulps,—"spunk of white birch," which is found on birch-trees that have been dead for some time. It is perfectly white, and its porosity favors its use. Coated with Canada balsam it can be placed directly over the pulp, and it has sufficient resistance to prevent its depression on the pulp by the presence of oxychloride, provided the latter is mixed to a proper consistence.

*Dr. J. Bond Littig*, New York City.—In regard to the use of sterilized sponge, the only advantage I see in that form of capping is its use on exposed pulps. In this pathological condition there is a slight secretion, and by placing the sponge over it is taken up. In general treatment the secretion oftentimes becomes putrescent and destroys the pulp under the capping; but in this method, the sponge being absorbent and itself thoroughly sterilized, we might look for some good results. That is the only advantage I can see in the use of the sponge.

The President then introduced Dr. J. N. Crouse, Chicago, Chairman of the Executive Committee of the Dental Protective Association, who spoke as follows:

### THE DENTAL PROTECTIVE ASSOCIATION.

I have been querying since I came to Boston whether I had not made a radical mistake in the formation of my pet scheme,—the Dental Protective Association,—in not first launching it in Boston in the Massachusetts Dental Society. It seems to me it would have been a very proper thing, as you will remember it was a hundred years ago when the first rebellion against oppression was demonstrated in Boston, when the fifty men, rebelling at the imposition of the foreign government, threw out the contents of the vessel and refused to submit any longer; and when, by a process of starvation, the English government expected to bring them to terms, you remember how the other parts of the country came to their rescue by aiding them with food that kept them from perishing until, finally, the end was accomplished and the independence of this country was secured.

It might have been proper to have withheld the formation of my Protective Association until this meeting, and yet the ground I have gone over and good I have accomplished I do not regret; only it might have been preferable to have brought it here first.

First, What is the object and what is this Protective Association of the United States? Heretofore, you will remember that when we wished to defend ourselves it has been by subscription, always costing the individual much more than the small sum that is asked here. After casting about it seemed necessary to form a permanent organization, and under competent legal advice we formed a corporate body under the laws of the State of Illinois, which required that we should have a board of directors. These are the responsible parties for the body. It leaves the members free from any harassment if such an attempt is made, leaving it for the board of directors to do the work and receive the brunt of the attack if it comes from the opposition. Now, all who have examined the plan of organization will see that the membership is on a basis of ten dollars. Each member signs the constitution and by-laws and pays ten dollars. In signing you agree to submit to a further assessment of ten dollars. There are enough men in the dental profession who will come into this organization to preclude the necessity of further assessments.

The object of the Protective Association is, first, to take care of what is generally admitted to be an imposition upon the dental profession,—the matter of the International Tooth Crown Company's patents. The International Tooth Crown Company, as you know, own several patents: I think I have the record of twenty-six in my satchel. It is true that many of these patents in themselves are harmless,—that is, they are harmless if you have any means of defence. The International Tooth Crown Company have a decision on one patent in their favor, and that is the gold bridge patent. In their suits, which were held in New York, that was the only patent in which they were sustained. The other patents this company have taken to the Supreme Court of the United States, where the suits are now pending. Besides this bridge, they have a large number of patents, many of which seem to me very amusing. They start out with patents, and name them as "preparation of roots for crowns," patents for "cutting off teeth," patents on "destroying the pulps,—driving it out." There is also a patent on filling the end of this root, as the patent describes, before there is any danger of inflammation or suppuration. I do not know whether it is the danger of suppuration you are to avoid or whether it is the cedar stick driven into the pulp cavity. They have a patent on freezing a tooth so it will not be sensitive; also another on cementing a pin or post into the root of a tooth. They have, in addition, a patent on filling teeth with some fibrous material; also many other patents covering crowns,—ten, twelve, or fifteen of them,—which would seem to cover all the crowns ever invented. These patents on the crowns, if held to be good, would certainly decide that the Logan, Bonwill, and various other forms of crowns are infringements of some of these patents. If we remain quiet and they are sustained, the probability is they will take up everything else they can buy. They will come around with a mule-team, and if one thing does not suit your case they have another, so that if they come across a man who is not doing bridge-work,—perhaps he is destroying nerves,—they could torment him and the dental profession, and no individual alone could contest these patents, because it is too expensive. Therefore the necessity of some concerted action by which this imposition can be stopped. It seemed feasible and right that each individual should give his part to the cost. The matter of ten dollars is a small sum of money as compared with the great amount of good that can come from it in other ways. The Protective Association offer in return for this money and this membership to take care of all suits,—guarantee to take care of them, furnish counsel and testimony, and relieve the individual dentist of all expense, bother, and trouble. If they are to be sued, all they have to do is to turn over the matter to the Protective Association and allow it to take care of the suit. This method of defence will make it very effectual. I heard of a remark made by one of the representatives of this company, which was, that the only thing they feared was that the dentists would organize a body and bring their strength in one mass. That was the only thing that could make trouble for the patentees. I am here, gentlemen, representing this Protective Association, with the belief that you will recognize it, and stand by each other.

The dental profession are more ignorant on the subject of law and patents than any other thing. You need not be surprised if the Supreme Court of the United States sustains the patents now before it in favor of the Crown Company. We rather expect it, although we hope it will not do so; but just as soon as we hear that the Supreme Court has gone against us, that is just the time when we will really begin our defence. If the company have the decision in their favor, then they will apply all through the United States for injunctions against the dental profession, because the profession, as a mass, have infringed their patents. When they do this, it is the object and aim of the Protective Association at that time to prepare a new record and try these cases over again on the testimony we have gathered. I have received a large number of drawings, and the names of the parties who are wearing crowns and bridges, in response to the circular I sent out. It is the purpose of the Protective Association to have a full and complete record ready to bring into court at any moment, in any place in the United States, to apply on the subject, and thus compel the International Tooth Crown Company to fight the battle over again, and fight it on the merits of their patents. Before that time comes there are suits now pending in the Supreme Court which the International Tooth Crown Company confidently expect to have decided in their favor. Mr. Offield, on the part of the Protective Association, says, "We must not be surprised, and must be ready for the result." If they continue this suit before the Supreme Court, that only brings the Low bridge up, so it leaves little chance for the profession to have a trial. But we can make them come into court and try the case over again. When the suit was tried in New York, of course it was new to the profession, and there was much evidence presented that was not pertinent. The responses of the profession were not very liberal, either, so it was with my meagre means they accomplished what they did in that suit; and when I speak of that in company with the record of the Protective Association, I do so wishing you to understand that it is not in the form of criticism; but we have been working a long time on this, and we have more to show up the next time.

It is the confident belief of the attorneys of the Protective Association that we have all the evidence necessary to fight every patent now owned by the International Tooth Crown Company, and Mr. Offield is not a man who would speak unadvisedly. I had expected to have him here and present the legal part of this matter to you in much better form than I can do it; but he is interested in some other patent suits, and was called suddenly home to Chicago.

Another subject of which the profession seem very "scary" is the thought of what their treatment will be providing the profession do not come up and give us evidence enough to win our suit. They say, "Suppose you do fight and lose,—then what?" Will they not be much harder on us? I want it understood that the company have established their rates of royalty. Any court will prevent them from increasing the royalty on the patents upon which they now demand a royalty of the dental profession; and it is a little surprising to see how far that company does intimidate the profession all over the country. There were a great many patentees before the first of January. Since that time there have not been so many. This Protective Association is not only to defend ourselves against the present imposition, but they may assail us where we cannot afford to go to law, and at a time when we cannot afford a permanent organization. We ought to have one thousand dentists in the Dental Protective Association before long, and should have five thousand before we leave off. That means fifty thousand dollars. We are under no expense except for our attorneys and printed matter to reach the profession. That will be lessened as they respond promptly. We are going to keep this thing going until we get there. I want you all to take home some fire with you, and kindle the fire about you. If we get this Protective Association fully organized, no man knows what benefits will come from it as a permanent organization. If any of you have been where you have not had a bank account, and have had a lot of bills hanging over you, and no money to pay them, you know that it is a mighty uncomfortable feeling; and if you have not been there I have. This movement will give the dental profession a bank account, and protection, whereby the strength of the dental profession can be brought together to take a stand on any proposition that is offered.

Further than this, we do not look for abuse all the time; but what could we do if we had the interest on fifty or one hundred thousand dollars, in the way of scientific work? We could employ experts to cultivate these microbes and tell us what they do and how they originate. It would be a great idea to have such men as Black and others, who on account of feeble health cannot practise, go into their laboratories and work out what the dental profession has not time and the ability to do.

I want to say just here that every member of the Massachusetts State Dental Society, and the friends who have not responded to the circular I mailed, should do so before they go home. Then I can feel that I have a friend here and there, and that the Protective Association extends all over the country. I will not need all of the money until February. After it numbers one thousand or fifteen hundred, the membership fee may be increased to twenty dollars or twenty-five dollars, so you had better hurry up and get in.

I do not want to take charge of your Society. I would suggest in your case that some of your live fellows should start around quietly while the proceedings are going on and get the members to sign, and take their ten dollars. When I get home I will forward a receipt, etc. If they have not ten dollars with them, get a piece of paper and put down their names, stating, "We join the Protective Association, and will forward our dues not later than the first of February or March, 1890."

There has been considerable said in the journals, and other places, as to what the International Tooth Crown Company was going to do with all of us fellows who are interfering with their game. I have had several letters myself. I have also been notified that I shall be held responsible for slander and libellous utterances; that they will commence suit against me for infringement of their patents, and for damages. I say I live in Chicago, and that they had better commence, and not talk too much. They have also threatened our treasurer in two or three letters, to which he has replied, "I do not become responsible for anything the Association writes, but I receive the funds here." They dictated a letter in which they wished him to retract some things in the circular letter in relation to the matter.

Then there is a suspicion in my mind that there are some others who are getting scared. I have been trying for some time to get some of the leading journals to give you the information that you ought to have from month to month. They say, "We have consulted our lawyer, and we may get ourselves in trouble." They are afraid; but I have offered to be responsible for their suits and damages. There is a little whispering around the offices of some of these journals that the Dental Protective Association will get too much power. I have had some of the dental dealers who run journals come to me and say, "Crouse, what else are you going to do? What are your plans hereafter?" I say, it is to work off what we have on hand first, and see what comes afterwards. They want to know what influence this Protective Association will have on their business. I do not believe the Association has any idea of interfering with their business. If there is any line of business that is imposing upon us, and we investigate and find it out, we may kick. I predict that the next warfare will be against the journals that do not do something for me. I have told them that the time has now come when they will have to choose whom they will serve, "the Lord or the devil."

DISCUSSION ON DR. CROUSE'S REMARKS ON THE DENTAL PROTECTIVE ASSOCIATION.

*Dr. J. N. Crouse*, in answer to a query as to how many members there were in the Protective Association, said, "In ten hours the International Tooth Crown Company would know how many we had if I told you. The responses we have are very good. It is twice as large as any organization of dentists existing in the world.

*Dr. George A. Maxfield*, Holyoke, Massachusetts.—I understand the names of all those joining the Association will be kept secret?

*Dr. J. N. Crouse.*—No one but myself knows who belong. They are not prosecuting any of our members yet.

*Dr. Merriam.*—I move that a committee be appointed by the chair to go through the audience and solicit subscriptions while the discussion is going on.

Carried.

*Dr. G. L. Curtis*, Syracuse, New York.—Not long ago I was doing work under the Low patent, and by special agreement they came to my office and taught me their methods, and after teaching me they asked me to take out a license, which I refused to do. Some time elapsed, after which I was sued, or a suit was opened to get a judgment for infringing their patent. The New York dentists and attorneys drew up papers which were so strong that the International Tooth Crown Company did not wish to continue the suit. Mr. Alvord, being at my office two or three weeks ago, asked me what I was going to do. I replied, "Try the suit,"—that we were anxious and desirous of making this a test case. He asked me if

I was a member of the Protective Association, and I told him I was happy to say I was. He then said my suit was not the one he was going to contest. This is the point I wish to bring out. I believe it is our duty, and one we owe the profession, to support this Association, even though we never make a crown. Its force will be felt in the future, and it occurred to me that there are many here to-day who would be glad to contribute even though they never infringe a patent.

*Dr. George A. Maxfield.*—I want to give an incident of my experience with Mr. Alvord, not quite two years ago. You will remember that this Society, also the Connecticut Valley Society, took the cue from the New York men, and took subscriptions of five dollars to help defend the suits and bear the cost. Mr. Alvord came to my office a few days after the meeting and asked if I knew where the money went to. I stated it was in good hands. He said, "I want to tell you something. Dr. Gaylord, of New Haven, owes his lawyer for one-half his services: the money is going for this, and you had better inform the members of that." You can see that was a downright falsehood.

Another thing we want to take into consideration regarding this Dental Protective Association is the character of the men who have taken hold of it,—an evidence in itself of its stability. Not only this, but think of the time these men are giving for our benefit, to say nothing of the money they are spending. They leave their practice, as Dr. Crouse has, coming all the way from Chicago in order to present this matter before us, and give us the full benefit of the defence for simply the ten dollars.

*Dr. I. J. Weatherby.*—A remark recently made, which would imply that the money which was raised by pledges for the defence here in Boston of the rubber suits commenced against myself was not properly applied, I would like to correct. I know where every penny went to, and where five hundred dollars additional from my own private purse went. There was not enough money to pay the expenses of the court and lawyers, and five hundred dollars came out of my own pocket which has never been repaid.

*Dr. L. D. Sheppard*, Boston, Massachusetts.—The charge Dr. Weatherby refers to has been misinterpreted, and was understood by him to apply to the first case in which he was interested. The statement made by the former gentleman is true, nevertheless, of suits that were carried on against the Dental Vulcanite Company. There were multitudes of them; in fact, their method, as will be remembered by those who are old enough to go so far back, was a movement skilfully planned and carried out. The whole history of the contest with the Dental Vulcanite Company was one of strong oppression, with plenty of money, against feeble defendants, with little money, and, of course, the victory was always on the strong side. The first suit here in Boston was a weak one because the profession did not come to the support of the private men who dared risk their money in defending that suit. There is no one who knows anything about the suit but will say that the money was put to the right use. I say this in justice to Dr. Weatherby, for it was said at one time that it was not an honest suit: it failed from lack of means. The same result would follow with this new company, which is the same old company, as is well known. The same men are in this company, and their methods are the same. We are just as sure to be bound hand and foot by the International Tooth Crown Company as we sit here to-day, if we do not manfully, hand to hand, support this movement. I stated two years ago before the Massachusetts Dental Society, when I was settling with the International Tooth Crown Company,—at a time when I could not afford to do otherwise,—that if I saw an occasion in which I, being the defendant in any imposition practised upon the profession, had to depend upon the pecuniary support of the profession to help me pay my bills, I would settle with the company, pocket the imposition, and decline to be the defendant. I will never be an individual in a suit in which I expect the support of the profession. I say so, perhaps, with shame and sorrow, but there has never been a time, since the trouble with the Dental Vulcanite Company to the present time, that I have not given my help and labored to collect money in which others were willing to be the defendant.

I honor any man who will so far neglect his private practice and give his time, which is worth to him thousands of dollars, as to become the target for abuse and the instrument against which a powerful imposition aims its shafts. I bow to that man, and say that he is a braver and better man than I am. If the members of the Massachusetts Dental Association, numbering perhaps one hundred men, do not send one hundred subscriptions to Dr. Crouse, I shall be able to repeat what I said a year ago last December,—that I was ashamed of my profession. In the whole history of the contests, from the first contest against the Vulcanite Company to the present, in every action in which generosity was required of the profession, the rule has been that a dozen, fifty, or one hundred men came up to the mark when there should have been five or ten thousand! Just so sure as this movement meets with a feeble response from each member of the profession and the Society, and they withhold their support of ten dollars, just so sure will the time come when a suit against an obscure and poor man here, another suit against another man a little distance away, and so half a dozen in this State and half a dozen in another State, will produce an accumulation of judiciary decisions that will be so strong in favor of this Crown Company that its claims will be sustained in the courts, and we shall be bound hand and foot. It is a skilful part of warfare by which defenceless detachments, situated far apart, are taken one by one and conquered, until the whole foe is conquered.

I speak with some earnestness for various reasons. I was asked before this meeting if I would say something on this subject. Whatever I did individually for my own protection, at a time when there was no help and no organization, I have no hesitation in acknowledging. I should do it again under similar circumstances. Always when there has been money to be raised I have been on the side of the profession, and I am to-day, and I want to add my earnest request that you all be patriotic and give your support to a movement which, if it fails, will probably be the last attempt at defence from many impositions.

*Dr. H. C. Merriam.*—There is one point entering into the Vulcanite Company's suits which is different from the case of the Tooth Crown Company's. There we had one or two patents to fight,—Goodyear's and Cumming's. In the present case they have a multitude of patents, which gives them a powerful influence over the profession; moreover, up to the present time there has been no limit to their scope, and they have accumulated a vast number of inventions, which was not possible with the Rubber Company. You go to the clergyman or lawyer, and his services are at your command. Not so with the dentist, however, for he in this case will depend upon the will of others for the right to practise his profession: he cannot do this unless he has a license.

Those who want to take an honorable position in an honorable profession cannot afford to pass this opportunity by. It is a question of honor, gentlemen,—our personal liberty,—and if you are not taking part in it it is an individual shame. We must meet organization with organization, and a company must be formed that is to cover every part of this country, and gather just as minutely as in the Patent Office the records of our inventions and resources of our profession, that they may be known as the source from which our strength has come in every movement of this kind. We have no realization of this power of patents, what a sly thing it is to take our title from the profession.

*Dr. I. J. Weatherby.*—Why was it that the suit I mentioned failed? Do any of you know? There was no money.

*Dr. L. D. Sheppard.*—Josiah Bakon once told me that the vital mistake was the want of money.

*Dr. I. J. Weatherby.*—If we had had the money in thirty days that case would have been appealed, but some thought it a dead horse, and that was the end of it.

*Dr. W. X. Sudduth.*—With the permission of the Society I would like to speak of a personal matter.

Permission being granted, Dr. Sudduth proceeded to unfold the plans and purposes of the company publishers of the INTERNATIONAL DENTAL JOURNAL, which are well known to all of our readers and need not be repeated here. Regarding the relation of the JOURNAL to the Dental Protective Association, he said that before the movement was brought out Dr. Crouse and he had consulted in regard to the best methods to adopt, and that the INTERNATIONAL DENTAL JOURNAL had been pledged as the organ of the Dental Protective Association. We have spared no pains to bring the movement forward and urge the necessity of organization upon the part of the profession. Owned and controlled as our journal is by members of the profession, it cannot do otherwise than stand by any movement that the profession as a body may think wise to adopt. We therefore pledge the hearty support of the JOURNAL to the Dental Protective Association.

*Dr. Merriam.*—As one of the stockholders of the INTERNATIONAL DENTAL JOURNAL, I should say that in my opinion it would be time for the corporation to cease its existence if it did not uphold such a movement.

